# Checkpoint regulator B7x is epigenetically regulated by HDAC3 and mediates resistance to HDAC inhibitors by reprogramming the tumor immune environment in colorectal cancer

**DOI:** 10.1038/s41419-020-02968-y

**Published:** 2020-09-15

**Authors:** Yuxin Li, Yao Liu, Na Zhao, Xiaojun Yang, Yaqing Li, Fangzheng Zhai, Xingxing Zang, Wei Cui

**Affiliations:** 1 Department of Pharmacology, Shenyang Pharmaceutical University, Shenyang, PR China; 2 Center for Neuroscience, Medical College of Shantou University, Shantou, PR China; 3 Department of Microbiology & Immunology, Albert Einstein College of Medicine, New York, NY USA

**Keywords:** Cancer microenvironment, Cancer therapeutic resistance, Colorectal cancer

## Abstract

HDAC inhibitors are efficacious for treating lymphoma, but display limited efficacy in treating solid tumors. Here, we investigated the relationship between HDAC inhibitor resistance and the tumor immune environment in colorectal cancer. Our data indicated that among the investigated immune factors, B7x expression was enhanced in HDAC inhibitor-resistant colorectal cancer models in vitro and in vivo. In addition, gene manipulation results demonstrated that xenograft mice with tumors derived from a B7x-overexpressing CT-26 colorectal cancer cell line were resistant to HDAC inhibitor treatment. Notably, we found that there is a negative relationship between HDAC and B7x expression in both colorectal cancer cell lines and patients’ tumors. Furthermore, our data indicated that elevated expression of B7x was related to a poor prognosis in colorectal tumor patients. Interestingly, treatment with a specific inhibitor or siRNA of HDAC3, but not HDAC2, 6, and 8, resulted in obvious upregulation of B7x expression in colorectal cancer cells. In addition, our data showed that a cell line with high HDAC3 expression and low B7x expression had decreased enrichment of acetylated histone H3 in the promoter region of the gene encoding B7x. This pattern was reversed by addition of HDAC3 inhibitors. Mechanistically, we found that HDAC3 regulated B7x transcription by promoting the binding of the transcription activator C/EBP-α with the B7x promoter region. Importantly, our data indicated that an antibody neutralizing B7x augmented the response to HDAC inhibitor in the colorectal cancer xenograft model and the lung metastasis model by increasing the ratios of both CD4-positive and CD8-positive T cells. In summary, we demonstrated a role of B7x in HDAC inhibitor resistance and identified the mechanism that dysregulates B7x in colorectal cancer. Our work provides a novel strategy to overcome HDAC inhibitor resistance.

## Introduction

Histone deacetylases (HDACs) are important epigenetic regulators that remove acetyl groups from the N-acetylated lysine residues in the tail of histones and condense the chromatin structure to mediate gene silencing^1^. Multiple HDAC isoforms have been identified and classified into different groups (HDAC class I–IV). Increased expression of HDACs is associated with the development and progression of cancer via dysregulation of gene expression. Thus, HDACs are potential therapeutic targets for the treatment of cancer^1,2^. To date, five HDAC inhibitors (HDACi)—SAHA, FK228, belinostat, panobinostat, and chidamid—have been approved as anticancer drugs^3,4^. Although they have shown great promise, HDACi resistance is frequent and they have limited efficacy in treating solid tumors^3,5–7^. Therefore, there is an urgent need to explore the mechanisms underlying HDACi resistance and to develop mechanism-based therapeutic approaches.

In fact, several mechanisms are known to contribute to HDACi resistance, including elevated levels of thioredoxin induced by p21, which counteracts ROS-mediated DNA damage^8^, and increased levels of Bcl-2, which mediate apoptosis resistance^8,9^. Other mechanisms of resistance have also been identified recently, including increased drug efflux^10^, changes in chromatin and epigenetic enzymes^8,11^, and the activation of survival pathways, such as STAT3, MAPK, and PI3K^12–14^. In spite of these advances, it is still a challenge to develop strategies for overcoming HDACi resistance. Recent studies have demonstrated that the combination of immune checkpoint inhibitors and HDAC inhibitors shows promising efficacy in vitro and in vivo^15,16^. These findings suggest that immune regulation in the tumor microenvironment might also be involved in HDACi resistance.

Immune checkpoint molecules, including PD-L1/2, B7-1/2, B7-H3, B7x, VISTA, and Galectin-9, have been characterized as potent regulators of immune activation, and play a crucial role in the mechanisms that mediate tumor immune escape^17,18^. Here, we take colorectal cancer as an example to investigate the relationship between HDAC inhibitor sensitivity and immune checkpoint regulation. Our results indicated that B7x is upregulated by HDAC inhibitor treatment in vitro and in vivo, especially in HDAC inhibitor-resistant cells. Mechanistically, HDAC3 is a crucial regulator of B7x transcription by increasing the binding of the transcription activator C/EBP-α with the promoter region of the B7x gene. An anti-B7x antibody augments the response to HDAC inhibitor in a colorectal cancer model.

## Materials and methods

### Cell lines and cell culture

The human colorectal cancer cell lines LOVO, Colo-205, SW480, and SW620 were obtained from Cell Bank of the Chinese Academy of Sciences (Shanghai, CHN). Human colorectal cancer cell line HCT-116 and the mouse colorectal cancer cell line CT-26 were obtained from American Type Culture Collection (ATCC; Manassas, VA, USA). The mouse colorectal cancer cell line MC-38 was obtained from Department of Medicine (Oncology) in Albert Einstein College of Medicine(New York, USA). These cancer cells were routinely cultured in RPMI-1640 or MEM medium supplemented with 10% fetal bovine serum and maintained at 37 °C in a humidified incubator with 5% CO_2_.

### Compounds and reagents

The Pan-HDAC inhibitor SAHA, the HDAC1,2 inhibitor Romidepsin, the HDAC3 inhibitor RGFP966, the HDAC1,3 inhibitor ITF-2357, the HDAC6 inhibitor ACY-775 and the HDAC8 inhibitor PCI-34051 were obtained from MedChemExpress, USA. The primary antibodies against HDAC3, NF-κB, Lef-1, C/EBP-α, PARP, and β-actin were purchased from Cell Signaling Technology (Danvers, MA). The antibody B7x and HDAC1 used for immunohistochemistry were got from Cell Signaling Technology (Danvers, MA) and Sant Cruz Technology (Dallas, TX), respectively. The primary antibody against RFX-1 was obtained from Abcam Technology (Cambridge, MA). The mouse B7x antibody was generated by our lab (Albert Einstein College of Medicine). The HDAC siRNAs and C/EBP-α siRNA was from Life Technologies, USA.

### Immunohistochemistry

A tissue microarray with approved ethical document was provided from National Human Genetic Resources Sharing Service Platform(2005DKA21300). For immunostaining, primary antibodies were diluted 1:200 (HDAC1) and 1:50 (human B7x). Evaluation of the intensity of immunohistochemistry staining and the proportion of positively stained epithelial cells was done as previously described by two independent pathologist^19^.

### Cell viability assay

The in vitro cell viability was determined by MTT assay. The cells (5 × 10^4^ cells/ml) were seeded into 96-well culture plates. After overnight incubation, the cells were treated with various concentrations of agents for 48 h. The optical density of each well was measured at 570 nm with a Molecular Devices M5 Reader. The experiments were performed in triplicate, and results were plotted as the mean ± s.e.m.

### Transient transfection

Human C/EBP-α full-length cDNA was cloned into the pCMV expression vector. The pCMV-C/EBP-α (2 μg/μL) was transiently transfected into Colo-205 or SW480 cells by Lipofactamine 2000 (Invitrogen) according to the manufacturer’s instructions. Transfection efficiency was verified by western blotting.

### Quantitative RT-PCR analysis

Total RNA was isolated from cells using RNeasy Mini Kits (Qiagen) as described in the product insert. The RNA was reverse transcribed with RevertAid First Strand cDNA Synthesis Kits (Thermo) and PCR was done using iQ SYBR Green Supermix and the CFX96 Real-Time PCR Detection System (Bio-Rad). Primers used in this study are listed in Supplementary Table 1. The expression of each gene was determined using the 2^−ΔΔCT^ method. Results were normalized against GAPDH. All experiments were performed in triplicate, and results were plotted as the mean ± s.d.

### HDAC activity assay

The HDAC assay was conducted with a HDAC fluorescent activity assay kit (Biovison, USA) as we previously reported^19^. HDAC activity is shown as the mean ± s.e.m. of three experiments.

### Western blot analysis

The colorectal cancer cells were gathered after treatment for the indicated time periods. Western blotting was performed as previously described^20^. Briefly, mouse or rabbit primary antibodies and appropriate secondary antibodies were used to detect the designated proteins. The bound secondary antibodies on the PVDF membrane were reacted with ECL detection reagents (Pierce; Rockford, USA) and exposed to X-ray films. The experiments were performed in duplicate and results were normalized to the internal control β-actin.

### Chromatin immunoprecipitation (ChIP) assay

The ChIP Assay Kit was purchased from Beyotime Biotechnology (Shanghai, China). Colo-205 cells, SW480 cells and HDACi-treated SW480 cells were prepared for ChIP assays, which were performed according to the manufacturer’s instructions. Ac-H3 or C/EBP-α antibodies were used for immunoprecipitation. B7x promoter primers were used to PCR-amplify the DNA isolated by ChIP assay, and real-time PCR was performed to analyze the amplification products. The experiments were performed in triplicate, and results were plotted as the mean ± s.e.m. The sequences of qPCR primers are listed in Supplementary Table 1.

### Mouse colorectal heterotopic tumor study

To assess the response to the HDAC inhibitor SAHA, CT-26 cell lines stably expressing B7x were established. Control CT-26 [MSCV] and CT-26 [B7x] cells (5 × 10^5^/100 μl PBS per mouse), as confirmed by trypan blue staining, were subcutaneously injected into the right flank of 7–8-week-old wide-type BALB/c mice. When the average tumor volume reached 50 mm^3^, mice bearing CT-26 and B7x CT-26 tumors were randomly divided into two groups: the control group (saline only, *n* = 7) and the SAHA group (40 mg/kg/3 days, i.p.; *n* = 7). After 15 days, the mice were sacrificed and the tumors were excised. The mRNA was extracted and analyzed by real-time PCR.

To test the efficacy of the combined treatment (SAHA+anti-B7x Ab), CT-26 cells (5 × 10^5^/100 μl PBS per mouse) were subcutaneously injected into the right flank of 7–8-week-old wide-type BALB/c mice. When the average tumor volume reached 50 mm^3^, the mice were randomly divided into four groups: control group (saline only, *n* = 6), SAHA (40 mg/kg/3 days, i.p. *n* = 6), anti-B7x Ab (200 μg/mouse /2days, i.p. *n* = 6), and combination group (SAHA+anti-B7x Ab). Tumor size was measured once every 3 days with a caliper (calculated volume = shortest diameter^2^ × longest diameter/2). The body weight was also measured once every three days to assess gross toxicity. After 15 days, the mice were sacrificed and the tumors were excised and stored at −80 °C until western blot analysis. The protocol was approved by the Committee on the Ethics of Animal Experiments of the Shenyang Pharmaceutical University. The above mentioned animal study was blinding done by independent researcher.

### Mouse lung metastatic tumor study

To further investigate the efficacy of the combination therapy and its correlation with the immune environment, we established a lung metastatic tumor model. A total of 10^5^ B7x overexpressed CT-26 cells were intravenously injected into the tail vein of wild-type BALB/c mice in 100 μL of PBS to induce the pulmonary experimental metastasis model. After one week, the mice were randomly divided into four groups: control group (saline only, *n* = 6), SAHA (40 mg/kg/3 days, i.p.; *n* = 6), anti-B7x Ab (200 μg /mouse /2days, i.p. *n* = 6, Each mouse received a total of 1.4 mg antibody), and combination group (SAHA + anti-B7x Ab). After 15 days, the mice were sacrificed and the lungs were excised and the size and number of metastatic tumors was determined by stereomicroscope. In a subset of mice from each experiment, lung tumors were harvested and digested into single-cell suspensions with mechanical dissociation followed by enzymatic dissociation using a mouse tumor dissociation kit (Miltenyi Biotec) and a GentleMACS Dissociator, then analyzed by flow cytometry for tumor cell surface markers and immune cell phenotypes^21^. The protocol was approved by the Committee on the Ethics of Animal Experiments of the Shenyang Pharmaceutical University. The above mentioned animal study was blinding done by independent researcher.

### Statistical analysis

Differences between experimental groups were evaluated by one-way ANOVA with Turkey’s post-hoc test using the SPSS11.5 software package for Windows (SPSS, Chicago, IL). The correlation of linear regression analysis was performed with Pearson *r* test. The Chi-square test was used to analysis the correlation of protein expression of tumor tissues. Survival curves were constructed using the Kaplan–Meier method. Statistical significance was based on a *P* value of 0.05 (*P* < 0.05, two-tailed test).

## Results

### Resistance to the HDAC inhibitor SAHA is related to B7x induction in colorectal cancer

**Fig. 1:**
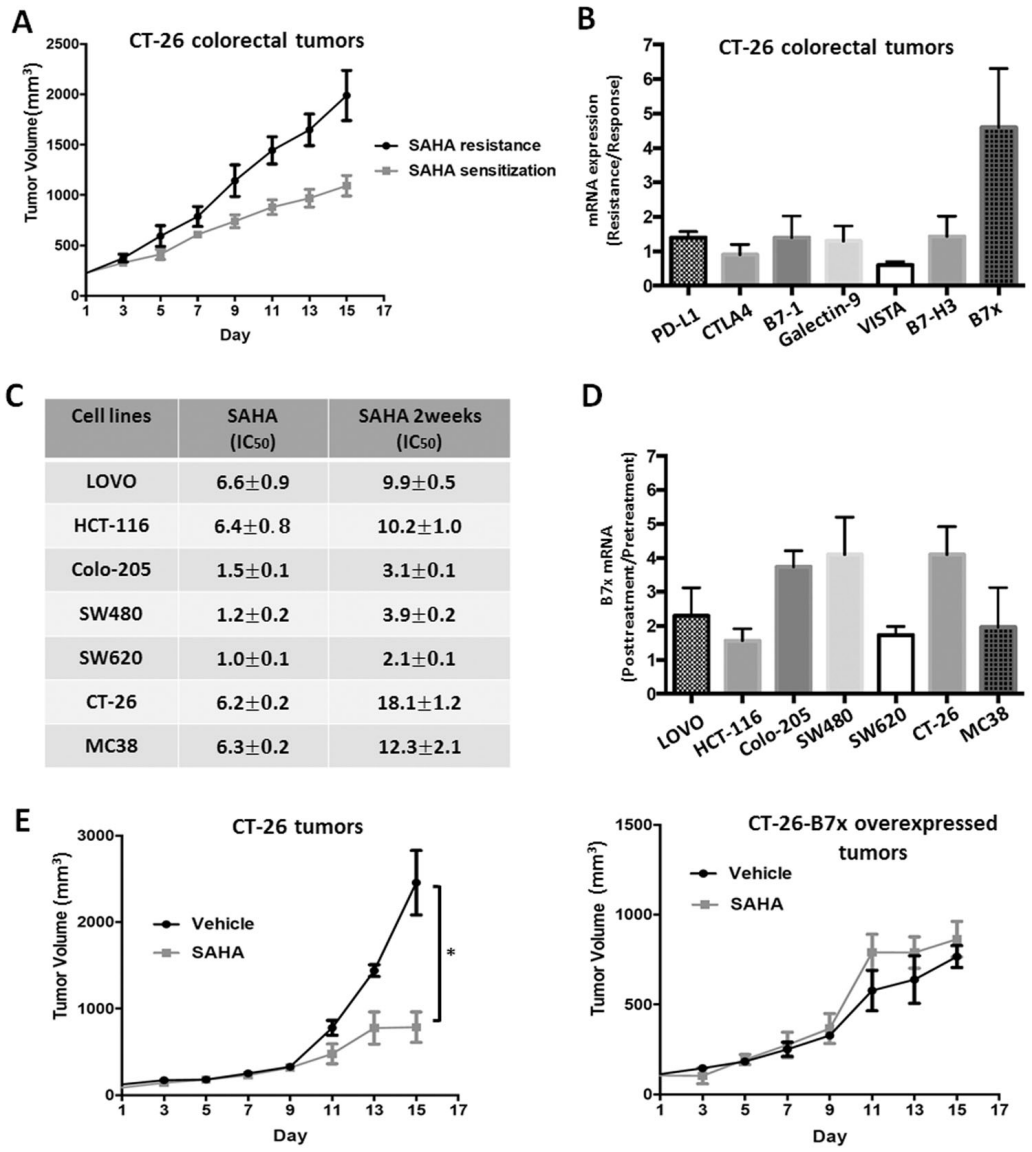
The relationship between B7x expression and HDAC inhibitor resistance in colorectal cancer. **a** Differential sensitivity of mice with CT-26-derived colorectal tumors to treatment with the pan-HDAC inhibitor SAHA. SAHA (40 mg/kg/3 days, i.p.) was given to tumor-bearing mice for 15 days. **b** The expression levels of mRNAs encoding immune factors in CT-26 colorectal tumor tissues. The mRNA was analyzed by real-time PCR. GAPDH was used as the loading control. **c** The viability of SAHA-pretreated and parental colorectal cancer cell lines after treatment with SAHA for 48 h. The IC_50_ (μM) is shown. **d** The expression level of B7x mRNA in SAHA-pretreated and parental colorectal cancer cell lines. **e** The antitumor efficacy of SAHA in the CT-26 tumor model (left) and the CT-26 model with B7x overexpression (right). All error bars are s.e.m. *P < 0.05, compared with vehicle control.

To mimic acquired HDACi resistance, we first established a CT-26 colorectal cancer heterotopic mouse model, in which tumor-bearing mice were treated with the pan-HDAC inhibitor SAHA for 4 weeks. As shown in Fig. 1a, the mice displayed differential sensitivity to SAHA. About half of the mice exhibited resistance to SAHA treatment, based on the larger tumor size. In order to further explore the relationship between HDACi resistance and immune checkpoint regulation, we measured the expression levels of mRNAs encoding the immune checkpoint molecules PD-L1, CTLA4, B7-1, B7-H3, B7x, VISTA, and Galectin-9, in both SAHA-responsive and -resistant tumor tissues. Our data indicated that only B7x mRNA was obviously increased in SAHA-resistant tumor tissues relative to SAHA-responsive tumor tissues. Next, we confirmed the SAHA-induced upregulation of B7x in seven colorectal cancer cell lines. The colorectal cancer cell lines were continuously treated with HDACi at 5 μM for 2 weeks. As shown in Fig. 1c, the IC_50_ values for SAHA in all seven colorectal cancer cells were significantly increased in the continuously treated cells. Real-time PCR data showed that the level of B7x mRNA expression was also increased to some extent in the continuously treated cells, which confirmed the in vivo data. To further confirm the role of B7x in SAHA resistance in vivo, we also established a mouse colorectal cancer model using a CT-26 cell line with overexpression of B7x. As shown in Fig. 1e, administration of SAHA significantly inhibited the growth of the CT-26 tumors, but could not block the growth of the B7x-overexpressing CT-26 tumors. The above results suggested that B7x is induced by SAHA and might be involved in the mechanism by which colorectal tumors acquire resistance to HDAC inhibitors.

### Expression of B7x is associated with HDAC activity and predicts a poor outcome

**Fig. 2:**
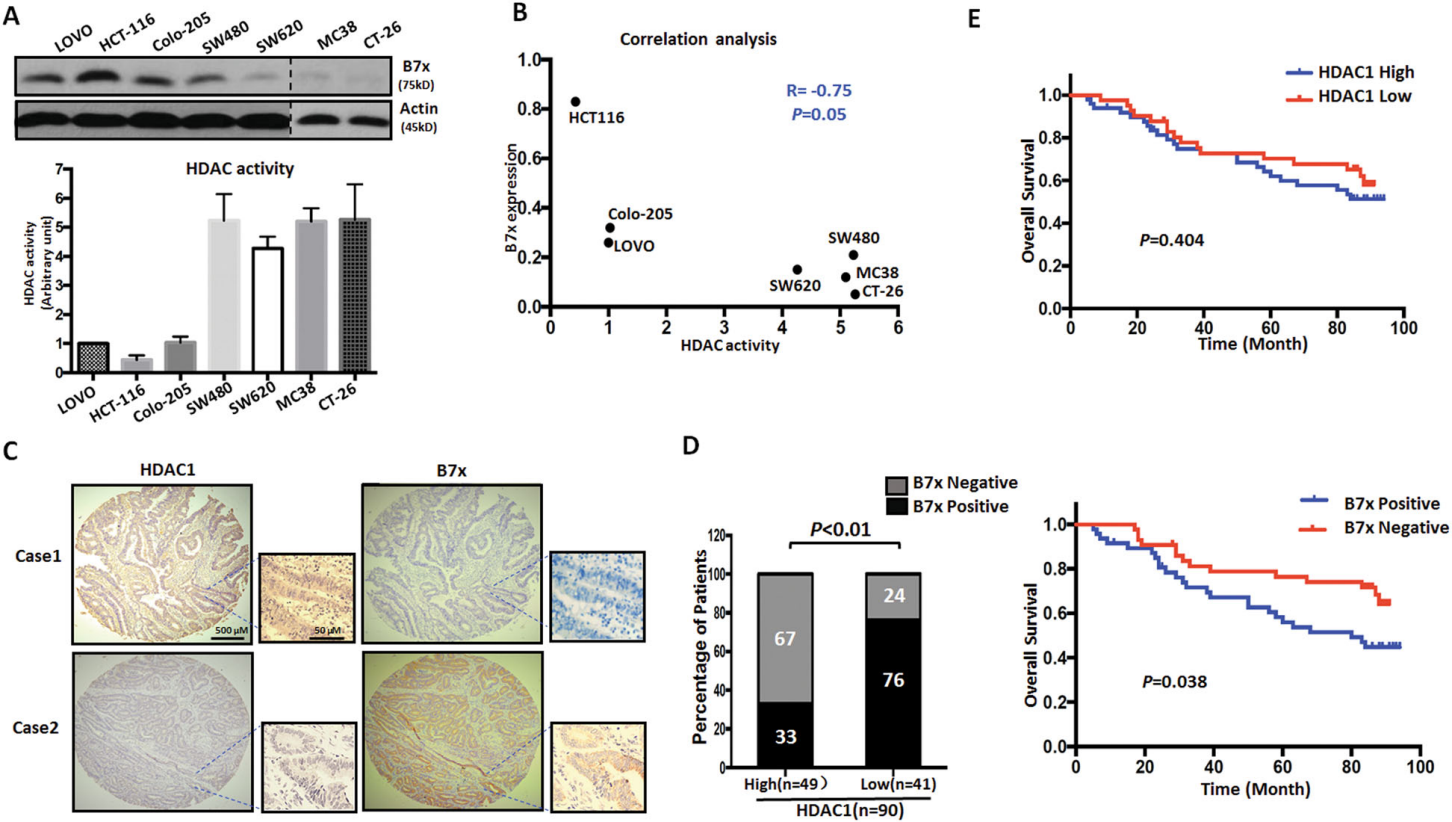
The negative relationship between HDAC and B7x in colorectal cancer cells and patients’ tumors. **a** B7x protein level and HDAC activity in colorectal cancer cell lines. The protein expression of B7x was measured by western blot, and HDAC activity was analyzed by microplate reader. β-actin was used as the loading control. **b** Correlation analysis of HDAC activity and B7x expression in colorectal cancer cell lines. **c** Representative images of HDAC1 and B7x expression in colorectal cancer tissues. Images are magnified ×40 or ×400. **d** The correlation between HDAC1 expression and B7x expression in colorectal cancer tissue specimens. **e** The prognostic significance of HDAC1 and B7x levels in colorectal cancer patients.

In order to investigate whether the induction of B7x was correlated with HDAC inhibition, we next examined the levels of B7x and HDAC activity in colorectal cancer cell lines. Our data showed that HCT-116, LOVO and Colo-205 cell lines expressed a relatively high level of B7x protein, and SW620 and SW480 cell lines expressed a moderate level, whereas MC-38 and CT-26 cells expressed a relatively low level of B7x (Fig. 2a). In contrast to the level of B7X protein, HDAC activity was higher in CT-26, SW480, MC-38, and SW620 cell lines, and was lower in HCT-116, LOVO and Colo-205 cell lines (Fig. 2b). The correlation analysis data showed that B7x expression is negatively associated with HDAC activity, with an *R* value of −0.75. Next, we confirmed this relationship between B7x and HDAC activity in tumor samples from 90 colorectal cancer patients. Our results indicated that 49 tumors (54.4%) displayed a relatively high level of HDAC1, which is the main functional isoform of HDACs. It should be noted that among the 49 tumors with increased HDAC1 expression, 33 tumors (67%) expressed a lower level of B7x (Fig. 2c, d). Statistical analysis of the data indicated that there was a negative correlation between B7x and HDAC1 levels in tumor tissue from colorectal cancer patients (*P* < 0.01, Fig. 2d). This confirmed the inverse relationship between HDAC activity and B7x expression. In addition, survival rate analysis indicated that the level of B7x was significantly correlated with the patients’ overall survival (*P* = 0.03, Fig. 2e, right). In contrast, the expression of HDAC1 was not related to the overall survival of the colorectal cancer patients (*P* = 0.35, Fig. 2e, left). These results suggest that B7x may be valuable as a prognostic marker. Taken together, these results strongly support the possibility that B7x expression is regulated by HDAC activity, and suggest that B7x plays an important role in the progression of colorectal cancer.

### The isoform HDAC3 specifically regulates the expression of B7x in colorectal cancer

**Fig. 3:**
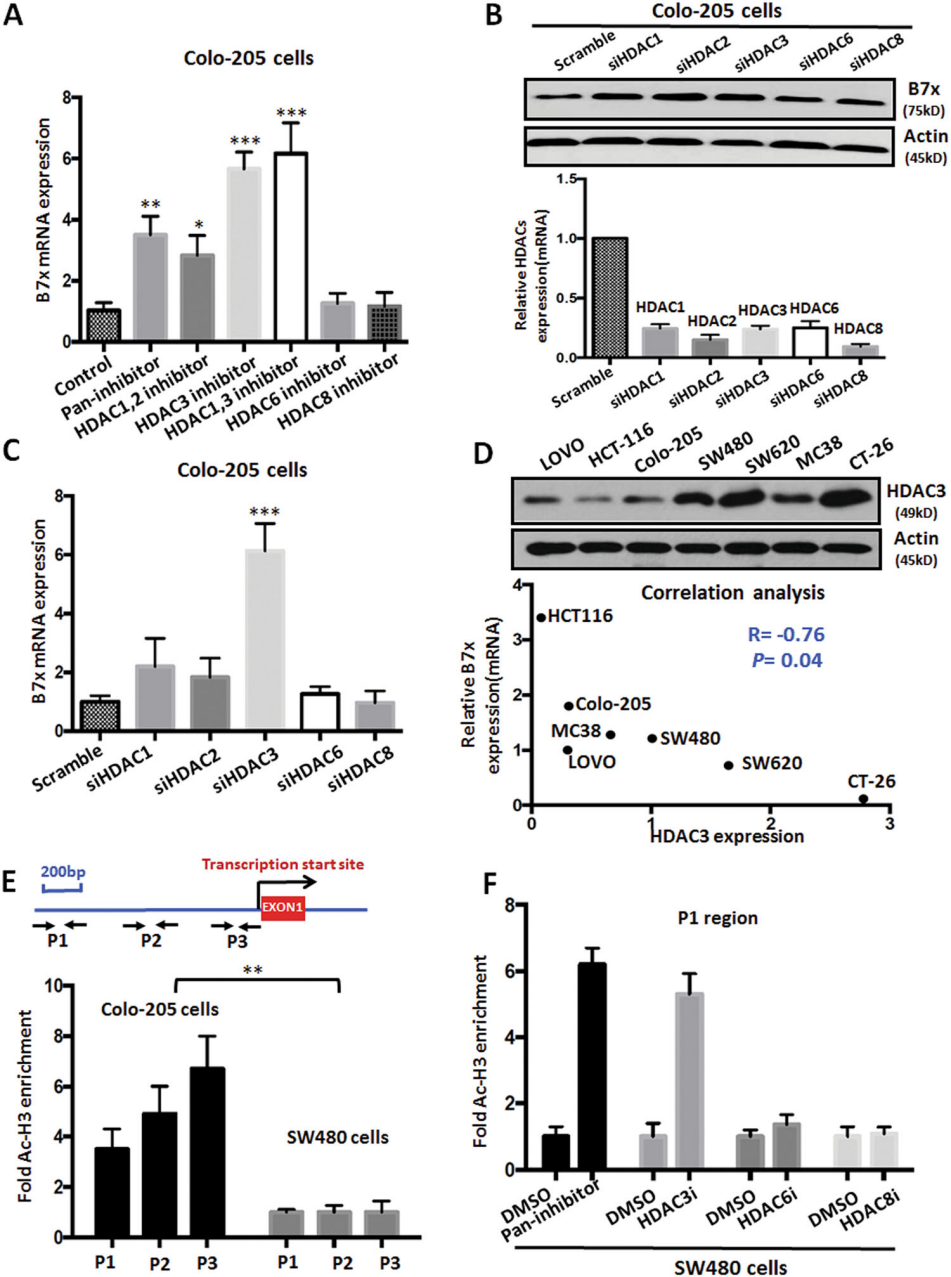
HDAC3 regulates B7x expression in colorectal cancer cells. **a** The expression of B7x mRNA in Colo-205 cells after treatment with various inhibitors (5 μM) for 48 h. The mRNA was detected by real-time PCR. GAPDH was used as the loading control. **b** The expression of B7x protein in Colo-205 cells after treatment with various specific HDAC siRNAs (100 nM) for 48 h(up). β-actin was used as the loading control. The efficacy after knockdown HDACs by specific siRNA were measured by real-time RT-PCR(down). Scramble siRNA was used as the control. **c** The expression of B7x mRNA in Colo-205 cells after treatment with various specific HDAC siRNAs (100 nM) for 48 h. GAPDH was used as the loading control. **d** The expression of HDAC3 protein and correlation with the B7x level in colorectal cancer cell lines. **e** The results of ChIP assays to detect the enrichment of Ac-H3 in three promoter regions (P1–P3) upstream of the B7x gene in Colo-205 and SW480 cells. The level of Ac-H3 in SW480 cells was considered as the control. **f** The enrichment of Ac-H3 in the P1 promoter region upstream of the B7x gene in SW480 cells after treatment with HDAC inhibitors (5 μM). DMSO-treated cells were used as controls. All error bars are s.e.m. **P < 0.01; ***P < 0.001, compared with control.

Different isoforms of HDACs exhibit different functions^22^, so we further investigated which isoform contributes to the regulation of B7x. Using real-time PCR, we assessed the expression level of B7x in Colo-205 cells after treatment with inhibitors of different HDAC isoforms. As shown in Fig. 3a, treatment with the pan-HDAC inhibitor SAHA or the HDAC1/2 inhibitor Romidepsin moderately induced expression of B7x mRNA in Colo-205 cells, whereas both the HDAC6-specific inhibitor ACY-775 and the HDAC8-specific inhibitor PCI-34051 did not induce the expression of B7x. Interestingly, the HDAC3-specific inhibitor RGFP966 and the HDAC1/3 inhibitor ITF-2357 both significantly upregulated the expression of B7x mRNA. In order to verify the specific role of HDACs in B7x regulation, we measured B7x expression in Colo-205 cells after transfection with various specific siRNAs for HDAC1, 2, 3, 6, and 8. The data showed that siRNA knockdown of HDAC3, but not other HDACs, led to significant upregulation of B7x at both the protein and mRNA levels (Fig. 3b, c). To further explore the relationship between HDAC3 and B7x, we investigated the expression level of HDAC3 in colorectal cancer cell lines, and found that there was a negative relationship between HDAC3 protein expression and B7x mRNA expression (*R* = −0.76, Fig. 3d). Next, to elucidate the underlying mechanism, we measured the accumulation of acetylated histone H3 (Ac-H3), a HDAC3 substrate, in both Colo-205 (relatively high B7x along with low HDAC3) and SW480 (relatively low B7x along with high HDAC3) cells. ChIP data showed that the accumulation of Ac-H3 was increased at promoter regions of B7x in Colo-205 cells but not in SW480 cells (Fig. 3e). In addition, we also found that the accumulation of Ac-H3 in SW480 cells was increased after treatment with the pan-HDAC inhibitor SAHA or the specific HDAC3 inhibitor RGFP966, but not with HDAC6 or HDAC8 inhibitors (Fig. 3f). Taken together, the above data demonstrate that the isoform HDAC3 specifically regulates the expression of B7x in colorectal cancer cells.

### HDAC3i and transcription factor C/EBP-α synergistically regulate B7x expression in colorectal cancer cells

**Fig. 4:**
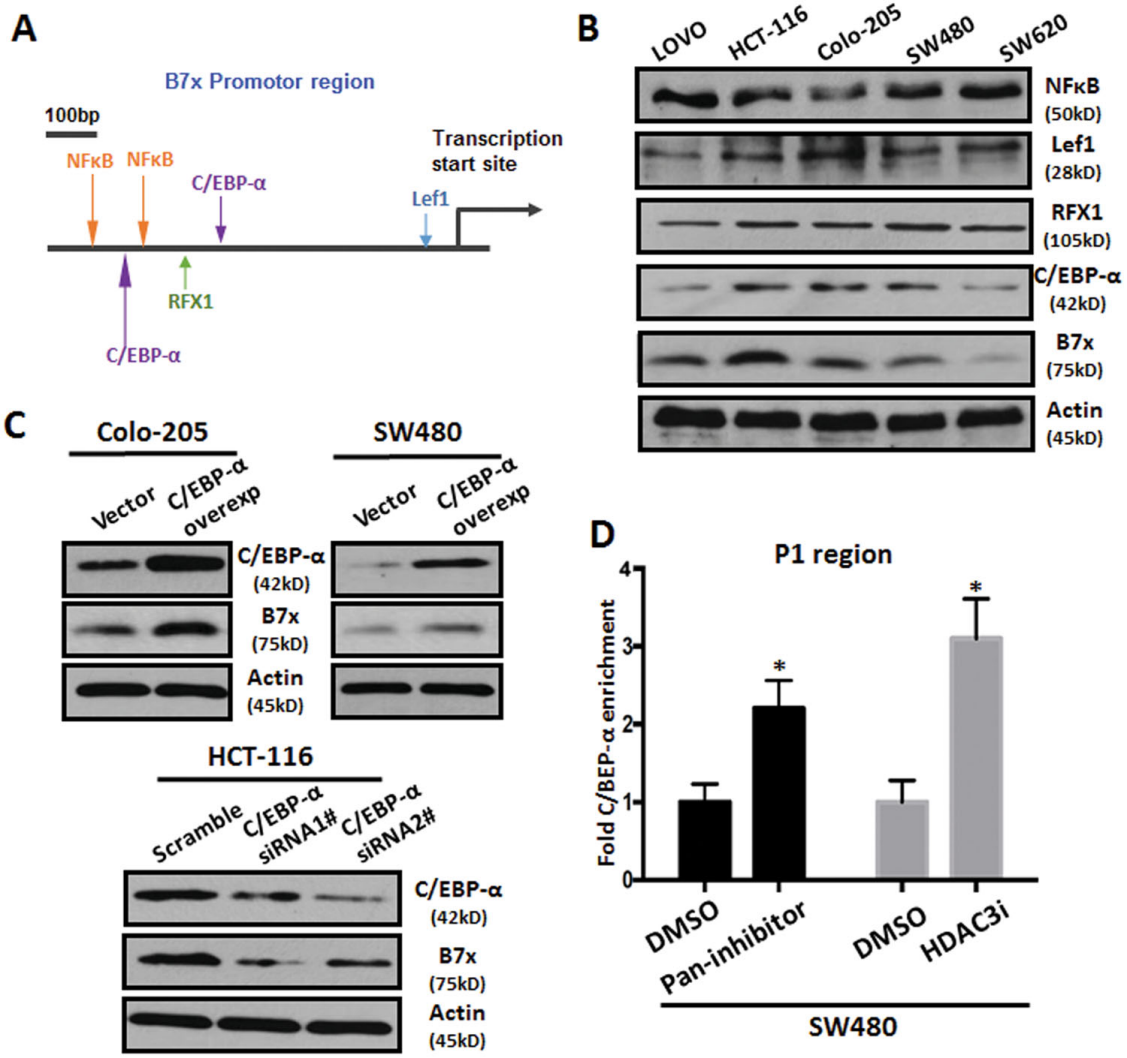
The transcriptional regulation characteristics of B7x in colorectal cancer cells. **a** The predicted transcription factor binding sites in the B7x promoter region. **b** The expression levels of the corresponding transcription factors in colorectal cancer cell lines. β-actin was used as the loading control. **c** The effect of gene manipulation of C/EBP-α on the expression of B7x protein in colorectal cancer cells. **d** The results of ChIP assays to detect the binding of C/EBP-α to the P1 region of the B7x promoter in SW480 cells after treatment with the pan-HDAC inhibitor SAHA (5 μM) and the HDAC3-specific inhibitor RGFP966 (5 μM) for 48 h. DMSO-treated cells were used as controls.

To further explore the mechanism by which HDAC3 regulates B7x, we analyzed the promoter region of B7x using the TRANSFAC transcription factor prediction system. The results indicated that four transcription factors—NF-κB, RFX-1, C/EBP-α, and Lef-1—had a higher matrix score (Fig. 4a). Next, we investigated the relationship between these four transcription factors and B7x in colorectal cancer cell lines. As judged by western blotting (Fig. 4b), only C/EBP-α displayed a positive correlation with B7x. The relationship between C/EBP-α and B7x was further confirmed by genetic manipulation. Our data showed that overexpression of C/EBP-α induced expression of B7x in both Colo-205 and SW480 cell lines, whereas knockdown of C/EBP-α resulted in the downregulation of B7x in HCT-116 cells (Fig. 4c). Importantly, we further confirmed that treatment with pan-HDACi or HDAC3i increased the binding of C/EBP-α to the P1 region of the B7x promoter, which includes the C/EBP-α binding site (Fig. 4d). In summary, the above results suggested that the transcriptional regulation of B7x was mediated by cooperation between HDAC3 and C/EBP-α.

### B7x neutralizing antibody sensitizes the heterotopic colorectal cancer mouse model to HDAC inhibitor treatment

**Fig. 5:**
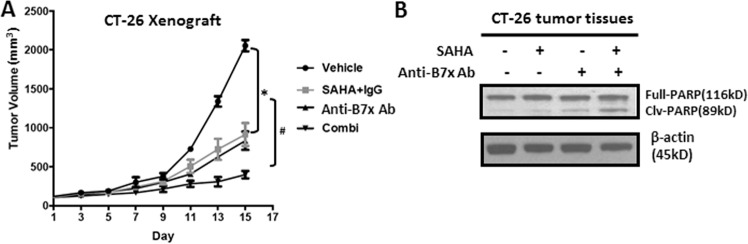
Effects of SAHA and/or Anti-B7x Ab on tumor growth and apoptosis in the CT-26 heterotopic mice model. **a** Tumor volumes were measured in CT-26 heterotopic mice treated with vehicle, SAHA+IgG, Anti-B7x Ab or the combination of SAHA+Anti-B7x Ab (Combi). **b** The level of PARP was measured in tumor tissues from the CT-26 mice. β-actin was used as the loading control. *P < 0.05, compared to vehicle control; ^#^*P* < 0.05, compared to vehicle and single treatment groups.

To investigate the therapeutic value of the above finding, we established the CT-26 heterotopic mouse colorectal cancer model. As shown in Fig. 5a, administration of the pan-HDACi SAHA for three weeks had moderate efficacy, with a tumor inhibition rate of 53.5%. On the contrary, single treatment with a B7x neutralizing antibody just yielded a weak antitumor efficacy, with a tumor inhibition rate of 27.8%. Notably, the combination treatment of SAHA and B7x neutralizing antibody significantly retarded tumor growth as compared to the single treatment and control groups. The tumor inhibition rate of the combination treatment was 80.6%. To further explore the underlying mechanism, we examined the apoptosis status of tumors from the four groups. As shown in Fig. 5b, western blot data showed that the cleaved PARP band was stronger in the SAHA and neutralizing antibody groups than in the control group, which suggests that apoptosis is enhanced by the single treatments. Among the four treatment groups, the combined treatment group displayed the strongest cleaved PARP band, which is consistent with the antitumor efficacy data. Taken together, our results showed the B7x neutralizing antibody sensitized the tumors to HDAC inhibitor in the colorectal cancer mouse model.

### The combination of B7x neutralizing antibody and HDAC inhibitor reduces metastasis in a mouse lung metastatic model

**Fig. 6:**
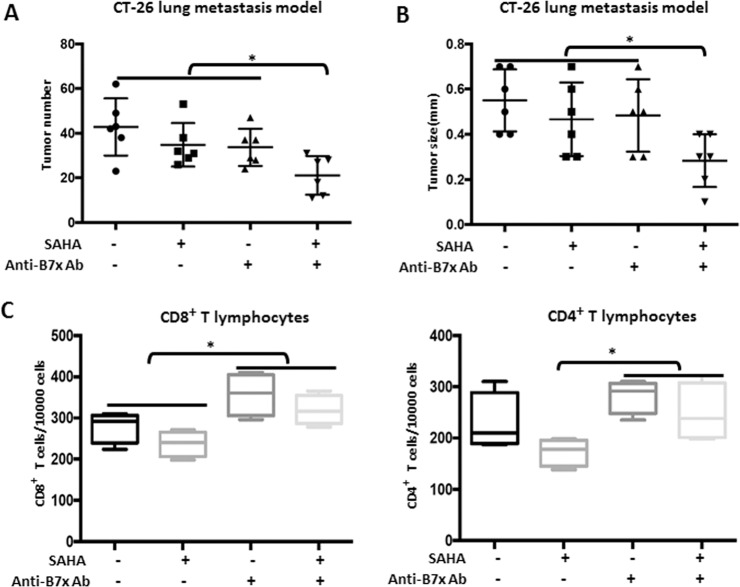
Effects of SAHA and/or Anti-B7x Ab on tumor growth and T-cell numbers in the B7x overexpressed CT-26 lung metastatic mice model. **a** The number of lung metastases and **b** the size of the metastases were measured in B7x overexpressed CT-26 metastatic mice treated with vehicle, SAHA+IgG, Anti-B7x Ab or the combination of SAHA+Anti-B7x Ab. **c** CD8^+^ and CD4^+^ T-lymphocyte counts were measured by flow cytometry. *P < 0.05, compared to the SAHA single treatment group or vehicle control group.

As metastatic disease represents the major challenge in the clinical treatment of colorectal cancer^23^, we next established a B7x overexpressed CT-26 mouse lung metastasis model by intravenous injection and tested the efficacy of the single treatments and the combined treatment. As shown in Fig. 6a, b, single administration of B7x neutralizing antibody and SAHA achieved marginal efficacy in reducing the number and size of lung metastases, whereas the combined treatment had a significant effect. Next, we counted the number of CD8^+^ and CD4^+^ T cells infiltrating the metastatic B7x CT-26 tumors. To do this, we performed tumor section flow cytometric analysis on freshly isolated in vivo metastatic samples. Our results showed that, as compared with vehicle control group, there was a decreasing tendency, but without statistical significance, of the proportion of CD8^+^ and CD4^+^ T cells in SAHA treated group. Notably, the proportion of CD8^+^ and CD4^+^ T cells was significantly increased in tumors from mice administered with the B7x neutralizing antibody alone and with the combined treatment as compared with SAHA treated group (Fig. 6c). The above data suggested that the B7x neutralizing antibody synergized with HDACi in the colorectal cancer model through regulation of the tumor immune microenvironment.

## Discussion

Inhibitors targeting HDACs have been used in the treatment of hematological malignancies, including peripheral T-cell lymphoma and cutaneous T-cell lymphoma^24^. However, most of the clinical trials on HDAC inhibitors showed that these drugs only have a limited efficacy in solid tumors. This suggests that there might be an intrinsic resistance to HDAC inhibitors in solid tumors, but not in hematological malignancies. Recently, a large number of published papers have reported that the tumor immune microenvironment plays a crucial role in the regulation of malignant behavior, including drug resistance, in solid tumors^25,26^. Here, we took colorectal cancer as an example to investigate the relationship between HDACi resistance and tumor immune factors. Our data showed that expression of the immune factor B7x was enhanced after HDAC inhibitor treatment in vitro and in vivo. Clinical immunohistochemistry demonstrated that there was a negative correlation between B7x and HDAC levels in tumor samples from colorectal cancer patients, which further confirmed the relationship between B7x and HDAC. Mechanistically, we found that HDAC3, an HDAC isoform, regulated B7x transcription by promoting the binding of the transcription activator C/EBP-α with the promoter region of the B7x gene. Importantly, our data indicated that a B7x neutralizing antibody augments the response to HDAC inhibitor in a mouse colorectal cancer model.

B7x, also known B7-H4 or B7S1, is a member of the B7 family of immune regulators. Molecules in this family can stimulate or inhibit T cells^27^. It has been reported that B7x has an immunosuppressive role which leads to the inhibition of CD4^+^ and CD8^+^ T cells^27,28^. Furthermore, several studies indicated that B7x is overexpressed in multiple tumors and is often related to a poor outcome for patients^27,28^. As a co-inhibitory molecule, B7x is known to be associated with TCR-mediated T-cell proliferation, cell-cycle progression, and IL-2 production^28,29^. However, the role of B7x in drug resistance has not yet been elucidated. Here, we present the first evidence that the expression of B7x, but not other co-inhibitory molecules, is induced by HDAC inhibitor treatment and is associated with HDAC inhibitor resistance in colorectal cancer. Clinical study showed that B7x expression was negatively correlated with the patients’ overall survival, which further demonstrates the crucial role of B7x in colorectal cancer. Taken together, our results indicate that the co-inhibitory immune molecule B7x not only contributes to tumor growth, but is also involved in drug resistance. This information suggests a novel combination strategy against HDAC inhibitor resistance.

HDAC3, a class I HDAC isoform, has been shown to play an important role in the processes of apoptosis, cellular progression and DNA damage repair, which are often dysfunctional in cancer^30^. Furthermore, HDAC3 is overexpressed in a variety of cancers, including colorectal cancer, and therefore it is regarded as a promising target for cancer^30,31^. Here, we found that the HDAC3 is the main HDAC isoform that contributes to regulation of B7x expression. Knockdown or inhibition of HDAC3 results in the downregulation of B7x in colorectal cancer cells, suggesting the potential of HDAC3 as target for regulating the tumor immune microenvironment. Our data showed that mechanistically, HDAC3 synergistically cooperates with transcription factor C/EBP-α to regulate the expression of B7x. Interestingly, a recent study showed that HDAC3 regulates another co-inhibitory molecule, PD-L1, in lymphoma^32^. Therefore, HDAC3 might be a crucial epigenetic enzyme affecting the tumor immune microenvironment.

In clinical trials of HDAC inhibitors in patients with solid tumors, resistance to the HDACi usually leads to the failure of the trial^3^. Here, our results indicated that a B7x neutralizing antibody sensitized colorectal tumors to HDAC inhibitor treatment in vivo, and the combination of B7x neutralizing antibody and HDAC inhibitor showed enhanced suppression of metastasis in a mouse lung metastatic model. Notably, a recent study showed that a combination of romidepsin, a FDA-approved inhibitor of HDACs, and PD-1 blockade significantly inhibited tumor growth by enhancing the activation of tumor-infiltrating T cells in lung cancer^33^. Also, several studies have shown that the expression of co-inhibitory molecules, including PD-1, PD-L1, and CTLA4, was increased in cells resistant to chemotherapeutic or molecular targeting agents^34,35^. In addition, it has been demonstrated that the combination of checkpoint inhibitors with chemotherapeutic/molecular targeting agents can reverse drug resistance^36^. Therefore, treatments targeting the co-inhibitory molecules may be useful for reversing drug resistance in solid tumors.

In summary, our study showed that the elevated B7x expression in colorectal cancer may mediate resistance to HDACi treatment. Our findings suggest that B7x may be a useful indicator of HDACi resistance in colorectal cancer. Mechanistically, inhibition of HDACs, especially the HDAC3 isoform, leads to the epigenetically mediated upregulation of B7x through synergistic cooperation with transcription factor C/EBP-α. Importantly, we found that a B7x neutralizing antibody sensitized colorectal cancer cells to HDAC inhibitor treatment both in vitro and in vivo. Therefore, our work identifies an epigenetically regulated pathway, HDAC3/C/EBP-α/B7x, which is responsible for HDAC inhibitor resistance in colorectal cancer, and also provides a promising therapeutic approach to reverse HDAC inhibitor resistance.

## Supplementary information

Supplementary Table 1
