# Supplementary material for: Checkpoint regulator B7x is epigenetically regulated by HDAC3 and mediates resistance to HDAC inhibitors by reprogramming the tumor immune environment in colorectal cancer
Source: Cell Death Dis. 2020 Sep 15;11(9):753. doi: 10.1038/s41419-020-02968-y (PMC7493945; doi:10.1038/s41419-020-02968-y)
Supplement: Supplementary file 1 — Supplementary Table 1 [file 41419_2020_2968_MOESM1_ESM.docx]

**Supplementary table1**

Supple Tab.1 Realtime RT-PCR primer sequence

| Gene | Sequence | |  |
| --- | --- | --- | --- |
|  | Forward | Reverse |  |
| PD-L1(m) | GCTCCAAAGGACTTGTACGTG | TGATCTGAAGGGCAGCATTTC | |
| PD1(m) | GCACCCCAAGGCAAAAATCG | CAATACAGGGATACCCACTAGGG | |
| CTLA4(m) | TAGTGGGTATCCCTGTATTGCTG | CTTCTCTCGTCCCTGGAAGTC | |
| B7-1(m) | GCAGGATACACCACTCCTCAA | AAAGACGAATCAGCAGCACAA | |
| Galectin-9(m)  VISTA(m) | TTACTGGACCAATCCAAGGAGG  CTCCTTGCTATTTTCCTGGCTG | AGCTGTTCTGAAAGTTCACCAC  TATGGGCCGGTGTTCTTTGC | |
| B7-H3(m)  B7x(m) | GGACCTACGTCCAGGGAACAT  TGTTACATCCGCACCTCAAAAG | TGGTCACATTGCCAGTCAAGG  TCGCAGCGTAAACTCTCTGAA | |
| B7x(h) | TTCAAAGAAGGCAAAGATGAGC | GTCTCTGAGCTGGCATTATAGT | |
| HDAC1(h) | CATCGCTGTGAATTGGGCTG | ACCCTCTGGTGATACTTTAGCAG | |
| HDAC2(h) | ATGGCGTACAGTCAAGGAGG | TGCGGATTCTATGAGGCTTCA | |
| HDAC3(h) | AATTGCCTCTGGCTTACCTCC | TCTGGGATTGTGTGAACGCC | |
| HDAC6(h) | AGGTCGCCAGAAACTTGGTG | TGCCTGGTTGTGGTGGAATC | |
| HDAC8(h) | CGCTGGTCCCGGTTTATATCT | AGATGCTTCATCTCTCATCTGCT | |
| β-actin(m) | CTCCATCCTGGCCTCGCTGT | GCTGCTACCTTCACCGTTCC | |
| B7x P1 | TCTTGCTTTGTTGCCCATCG | TCTTGAGCCTAGCAGTTCGA | |
| B7x P2 | CACATAGACCCGCCTCTCAT | CCTAGTGTTTGCGTATGGGC | |
| B7x P3 | TACAGCTCCCTTCCACAGTC | AACTGGCTGTGCAGAATTCC | |
